# Shibi Tea (*Adinandra nitida*) and Camellianin A Alleviate CCl_4_-Induced Liver Injury in C57BL-6J Mice by Attenuation of Oxidative Stress, Inflammation, and Apoptosis

**DOI:** 10.3390/nu14153037

**Published:** 2022-07-24

**Authors:** Ruohong Chen, Yingyi Lian, Shuai Wen, Qiuhua Li, Lingli Sun, Xingfei Lai, Zhenbiao Zhang, Junquan Zhu, Linsong Tang, Ji Xuan, Erdong Yuan, Shili Sun

**Affiliations:** 1 Tea Research Institute, Guangdong Academy of Agricultural Sciences/Guangdong Key Laboratory of Tea Resources Innovation & Utilization, Guangzhou 510640, China; chenruohong@tea.gdaas.cn (R.C.); wenshuai@tea.gdaas.cn (S.W.); liqiuhua@tea.gdaas.cn (Q.L.); sunlingli@tea.gdaas.cn (L.S.); laixingfei@tea.gdaas.cn (X.L.); zhangzhenbiao@tea.gdaas.cn (Z.Z.); 2 School of Food Science and Engineering, Guangdong Province Key Laboratory for Green Processing of Natural Products and Product Safety, Engineering Research Center of Starch and Plant Protein Deep Processing, Ministry of Education, South China University of Technology, Guangzhou 510641, China; lyylianyingyi@163.com; 3 Guangdong Society of Plant Protection, Guangzhou 510640, China; 13535904444@163.com; 4 Taihongyuan Agriculture Co., Ltd., Xinyi, Maoming 525000, China; lisotong@sjbutton.com; 5 Hospital of South China University of Technology, Guangzhou 510641, China; xuanji@scut.edu.cn

**Keywords:** *Adinandra nitida* (Theaceae), Camellianin A, liver injury, oxidative stress, inflammation, anti-apoptosis

## Abstract

Liver injury is a significant public health issue nowadays. Shibi tea is a non-*Camellia* tea prepared from the dried leaves of *Adinandra nitida*, one of the plants with the greatest flavonoid concentration, with Camellianin A (CA) being the major flavonoid. Shibi tea is extensively used in food and medicine and has been found to provide a variety of health advantages. The benefits of Shibi tea and CA in preventing liver injury have not yet been investigated. The aim of this study was to investigate the hepatoprotective effects of extract of Shibi tea (EST) and CA in mice with carbon tetrachloride (CCl_4_)-induced acute liver injury. Two different concentrations of EST and CA were given to model mice by gavage for 3 days. Treatment with two concentrations of EST and CA reduced the CCl_4_-induced elevation of the liver index, liver histopathological injury score, alanine aminotransferase (ALT), and aspartate aminotransferase (AST). Western blotting and immunohistochemical analysis demonstrated that EST and CA regulated the oxidative stress signaling pathway protein levels of nuclear factor E2-related factor 2 (Nrf2)/heme-oxygenase-1 (HO-1), the expression of inflammatory cytokines, the phosphorylated nuclear factor-kappaB p65 (p-NF-κB)/nuclear factor-kappaB p65 (NF-κB) ratio, the phospho-p44/42 mitogen-activated protein kinase (p-MAPK), and the apoptosis-related protein levels of BCL2-associated X (Bax)/B cell leukemia/lymphoma 2 (Bcl2) in the liver. Taken together, EST and CA can protect against CCl_4_-induced liver injury by exerting antioxidative stress, anti-inflammation, and anti-apoptosis.

## 1. Introduction

The liver is a key controller of many physiological processes by mediating the synthesis and metabolism of endogenous compounds and participating in biological functions such as storage of liver glycogen, synthesis of secretory proteins, bile secretion, regulation of hematopoiesis, immune responses, and the metabolism of many existing exogenous compounds, including drugs, alcohols, and toxins [1,2]. The liver has a unique ability to regenerate and can fully recover from the most severe nonrecurrent conditions. However, a variety of conditions, including viral hepatitis, non-alcoholic fatty liver disease, chronic alcohol abuse, and long-term drug use, can lead to persistent liver injury, resulting in liver scarring and cirrhosis, which eventually progress to dysfunction and, later, hepatocellular carcinoma [3]. With the deterioration of the environment and the abuse of chemicals, liver injury has become a widespread disease with high morbidity [4].

Reactive oxygen species (ROS) are perceived to be molecular secondary messengers and play an important role in cell signal transduction and physiological processes [5]. However, when ROS are not cleared promptly, they can lead to intracellular protein, lipid, and nucleic acid destruction during pathological processes [6]. Due to the liver tissue usually being susceptible to pathological cascades of oxidative stress, inflammatory apoptotic response and excessive ROS generation have been postulated to trigger the formation of liver injury [7]. CCl_4_ is a chemical widely used in the laboratory to induce experimental acute liver injury, and its toxicity is mainly due to the generation of ROS [8] and the p-NF-κB [9], then leading to organ damage. The natural active ingredient flavonoids were found to have some hepatoprotective effects in experimental liver injury models in animals [10]. Silymarin is the most well-studied hepatoprotective flavonoid, which showed good hepatoprotective effects in CCl_4_-induced experimental liver injury models and has been applied in the treatment of liver injury [11]. Therefore, the exploration of safe, natural, effective efficacious flavonoid fractions of plant origin with hepatoprotective activity in a model for liver injury induced by intraperitoneal injection of CCl_4_ is essential to develop measures to elucidate the mechanism of hepatoprotective action.

Shibi tea, also known as Shiya tea, is a traditional non-*Camellia* Chinese tea prepared from *Adinandra nitida* leaves, which are high in flavonoids. Shibi tea is a unique wild tea that has been used as a health tea and herbal medicine in Southeast Asia, including China, for hundreds of years [12]. It is mainly grown in the cool, moist, and high-altitude (above 500 m) cliffs in southern China, and in recent years, it has also been cultivated on a large scale in Guangdong and Guangxi [13]. Shibi tea is not only tasty and sweet, but also rich in flavonoids (up to 28.4%), and it has been found to have antioxidant and antibacterial effects and prevent peptic ulcers [14,15]. Shibi tea is one of the plants with the highest flavonoid content. Among them, CA, a flavonoid glycoside with apigenin as its parent nucleus, is its main flavonoid (nearly 60%) [16]. However, the advantages of Shibi tea and CA in the prevention of liver injury are still unknown. In this study, we successfully established CCl_4_-induced liver injury in C57BL-6J mice and explored the anti-inflammatory, anti-apoptosis, and antioxidative effects of EST and the main flavonoid, CA, in repairing acute liver injury. We have established the hepatoprotective effects of Shibi tea and the functional ingredient CA in vivo. Consequently, we propose that Shibi tea might serve as a functional beverage with hepatoprotective effects, and emphasize CA as a novel natural plant-souse hepatoprotective ingredient that plays a significant role in the alleviation of liver injury.

## 2. Materials and Methods

### 2.1. Chemicals and Reagents

Shibi tea was obtained from Zhengqi Agricultural Development Co., Ltd. in Yingde, and from Taihongyuan Agriculture Co., Ltd. in Xinyi, Maoming, Guangdong, China. The EST was prepared and the component analysis of EST was realized in our previous study [17]. CA (HPLC ≥ 98%) was obtained according to our previous study [16]. The 4% paraformaldehyde (BL539A) was purchased from Biosharp (Anhui, China). 

### 2.2. Establishment of Murine Liver Injury Model

Male C57BL/6 mice (7 weeks old) were purchased from the Beijing Huafukang Bioscience Co. Ltd. (Beijing, China). The mice were housed at room temperature (22 ± 2 °C) with 60% ± 15% humidity on a 12 h light/dark cycle, with free access to deionized water and basic feed. The mice were acclimatized for 1 week, and then randomly divided into the following seven groups (*n* = 7 each): control group (Control), untreated CCl_4_ model (Model), CCl_4_ + 100 mg/kg silymarin (Positive), CCl_4_ + 30 mg/kg CA (L-CA), CCl_4_ + 100 mg/kg CA (H-CA), CCl_4_ + 200 mg/kg EST (L-EST), and CCl_4_ + 700 mg/kg EST (H-EST). Silymarin, EST, and CA were dissolved in a 0.5% CMC-Na solution. Except for the Control group, the acute liver injury model was induced by intraperitoneal injection of 0.2 mL/kg CCl_4_ (dissolved in maize oil) to each animal in the remaining groups, and the control group was injected with an equal amount of maize oil as a negative control. Two hours after intraperitoneal injection, gavage treatment was performed according to the dose administered to each group for three days. The Control and Model groups received the same volume of 0.5% CMC-Na solution.

### 2.3. Tissue Processing

Two hours after the 3-day treatment, the body weights of mice were measured. Then, the mice were anaesthetized with 40 mg/kg pentobarbital and euthanized by cervical dislocation. Whole blood was collected in heparinized tubes, and the sera were separated by centrifuging at 3000 rpm for 10 min. The wet weight of liver tissue was measured and collected for further analysis. The liver and spleen index were calculated as follows: liver or spleen index (%) = liver or spleen wet weight (mg)/mouse body weight (mg) × 100%. After weighing, the liver tissue was divided into two parts; the intact liver lobules were fixed in 4% paraformaldehyde solution for the preparation of liver tissue sections, and the remaining liver tissue was stored at −80 °C for subsequent analysis.

### 2.4. Biochemical Analysis

The serum levels of AST (C010-2-1) and ALT (C009-2-1) were measured using commercially available kits from Nanjing Jiancheng Bioengineering Institute (Nanjing, China) according to the manufacturer’s instructions. The liver tissues were fully homogenized by a homogenizer (OMNI Bead Ruptor 24) after adding saline in the ratio of weight (g) to volume (mL) of 1:9, and the homogenate was centrifuged at 4 °C for 10 min at 2500 rpm. The supernatant was taken and the protein content in the supernatants was measured using the Pierce BCA protein assay kit (Thermo VK312556). The ROS levels in liver were measured using an ELISA kit (MM-43700MA) purchased from Jiangsu Meimian (Yancheng, China). The levels of malondialdehyde (MDA) (A003-1-2), glutathione peroxidase (GPx) (H545-1-1), catalase (CAT) (A007-1-1), glutathione (GSH) (A006-2-1), and superoxide dismutase (SOD) (A001-3-2) in the liver were measured using specific assay kits according to the manufacturer’s instructions (Nanjing Jiancheng Bioengineering Institute).

### 2.5. Histological Evaluation

The fixed liver tissues were dehydrated with 70% ethanol for 24 h and further embedded in paraffin. Paraffin-embedded liver samples were sectioned (thickness approximately 2 μm) and stained with hematoxylin and eosin (HE stain) using a commercial kit (C0105S) from Beyotime (Shanghai, China) as the standard protocol. The sections were sealed with neutral gum and observed under a microscope (Olympus, Tokyo, Japan, 100 X), and a histopathological assessment was performed as previously described [18]. 

The severity of the liver injury is graded on a scale of 1 to 5 depending on the degree of cellular necrosis, coagulum, central area, and surrounding inflammatory infiltrate: a score of 0 indicates normal, 1 indicates very low (<1%), 2 indicates mild (1–25%), 3 indicates moderate (26–50%), 4 indicates moderate/severe (51–75%), and 5 indicates severe/high (76–100%) [19,20]. The injury score was averaged for each group of animals.

### 2.6. Western Blotting

The liver tissues (20 mg) were homogenized by a homogenizer in 180 µL Radio-Immunoprecipitation assay (RIPA) lysis buffer (P0013B, Beyotime, Shanghai, China) supplemented with 2 µL phenylmethanesulfonyl fluoride (PMSF). The tissue lysates further lysed were incubated on ice for 1 h and then centrifuged at 13,200 rpm at 4 °C for 5 min. The protein content was measured using the Pierce BCA protein assay kit (Thermo VK312556). An equal amount of protein per sample was collected and boiled for Western blotting analysis. Western blotting was performed as previously described [21]. The antibodies used in this work included Nrf2 (12721S, CST, Danvers, MA, USA), HO-1 (43966S, CST), β-actin (A1978, Sigma, MO, USA), NF-κB (8242S, CST), p-NF-κB (Ser536) (13346, CST), p-MAPK (3510, CST), Bcl2 (ab117115, Abcam, Cambridge, U.K.), and Bax (ab32503, Abcam). Positive signals were visualized using a chemiluminescence (ECL) analysis kit (170-5061, Bio-Rad, Hercules, CA, USA) and recorded with the Chemi Doc system (Bio-Rad, USA). The positive bands were quantified by densitometry using ImageJ software (Bethesda, Rockville, MD, USA) and normalized to the density of β-actin.

### 2.7. Immunohistochemical (IHC)

Immunohistochemical analysis was performed using the 3,3N-Diaminobenzidine Tertrahydrochloride (DAB) horseradish peroxidase color development kit (P0203, Beyotime) as previously described [22]. The sections were incubated with primary antibodies targeting tumor necrosis factor-α (TNF-α) (ab6671, Abcam, Cambridge, U.K.), interleukin-6 (IL-6) (SC-1265, SantaCruz, Dallas, TX, USA), Interleukin-1beta (IL-1β) (bs-0812R, Bioss, Woburn, MA, USA). After being mounted with neutral resin, the sections were observed under a light microscope (Olympus, Japan) and analyzed using the ImageJ software.

### 2.8. Statistical Analysis

The experimental results are presented as mean ± standard deviation (mean ± SD). Experimental data and graphs were statistically analyzed and plotted using GraphPad Prism 7 software for Windows (GraphPad Software Inc., San Diego, CA, USA). One-way analysis of variance (ONE WAY-ANOVA) was used to compare the significant differences between all groups, and all experiments were performed with at least three independent replications. *p* < 0.05 (* compared with the Model group/# compared with the Control group) and *p* < 0.01 (** compared with the Model group/ ## compared with the Control group) were considered statistically significant. 

## 3. Results

### 3.1. EST and CA Ameliorated the CCl_4_-Induced Liver Injury

**Figure 1:**
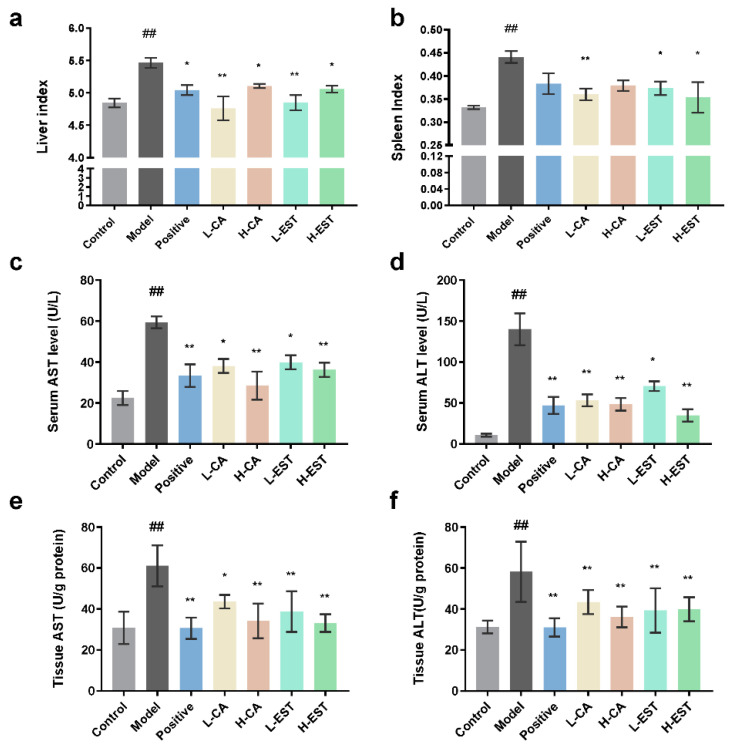
EST and CA ameliorated the CCl_4_-induced liver injury: (**a**) the liver index, (**b**) the spleen index of different groups, (**c**) serum AST, (**d**) serum ALT, (**e**) liver tissue AST, and (**f**) liver tissue ALT level of each group. Data are expressed as mean ± SD of at least three independent experiments (*n* ≥ 5). ## *p* < 0.01 compared with the control group; * *p* < 0.05, ** *p* < 0.01 compared with the model group.

As shown in Figure 1a,b, the liver and spleen indices of mice in the model group were significantly higher (*p* < 0.01) and decreased in mice treated with silymarin, CA, and EST. Among them, the liver index of L-CA and L-EST was comparable to that of the control group. To assess the effect of EST and CA on liver injury, the levels of AST and ALT in serum and liver tissues were detected, respectively. Compared with the control group, the AST and ALT of the model group were significantly increased (*p* < 0.01). Consistent with the gross observations, the AST (Figure 1c,e) and ALT (Figure 1d,f) levels of the mice treated with EST and CA were significantly reduced.

**Figure 2:**
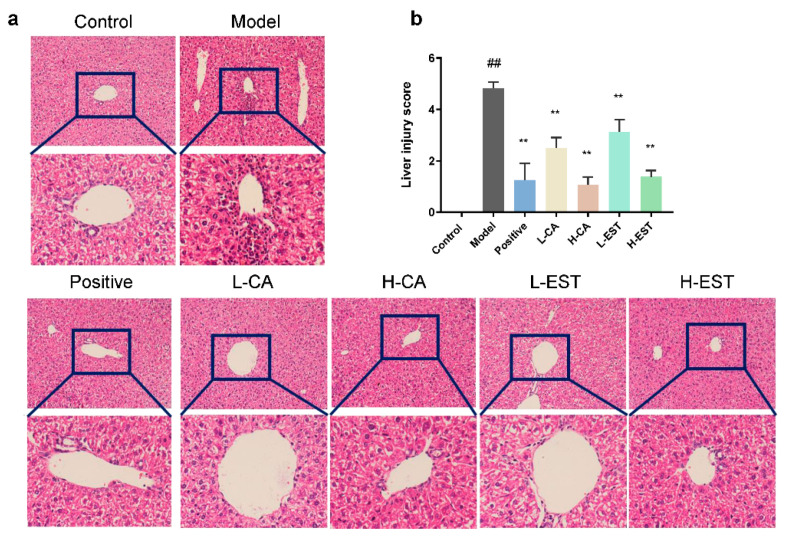
(**a**) Histopathological liver tissue (100×), (**b**) the liver injury score of mice in different groups. Data are expressed as mean ± SD of at least three independent experiments (*n* ≥ 5). ## *p* < 0.01 compared with the control group, and ** *p* < 0.01 compared with the model group.

Histological evaluation was performed to visualize the extent of liver tissue injury. As shown in Figure 2a, the hepatocytes of the control mice had normal morphology, regular and tight arrangement, and the structure of liver lobules was clear. After the CCl_4_ was induced, the liver tissues of mice in the model group showed obvious pathological changes, which were manifested by obvious inflammatory cell infiltration and disorder of hepatocyte arrangement. In comparison to the model group, treatment with the positive drugs silymarin, EST (200 and 700 mg/kg), and CA (30 and 100 mg/kg) alleviated the inflammatory cell infiltration induced by CCl_4_.. Furthermore, the liver injury score showed that EST and CA significantly reduced liver injury (*p* < 0.01) and restored normal cell morphology (Figure 2b). In addition, we detected the collagen fiber content of liver tissues in different treatment groups by using Masson trichrome staining. Masson staining of the model group showed increased collagen staining. Both CA and EST ameliorated the histopathological lesions in the CCL4-induced model group of mice (Figure S1).

### 3.2. EST and CA Decrease the CCl4-Induced Oxidative Stress by Activating the Nrf2/HO-1 Pathway

**Figure 3:**
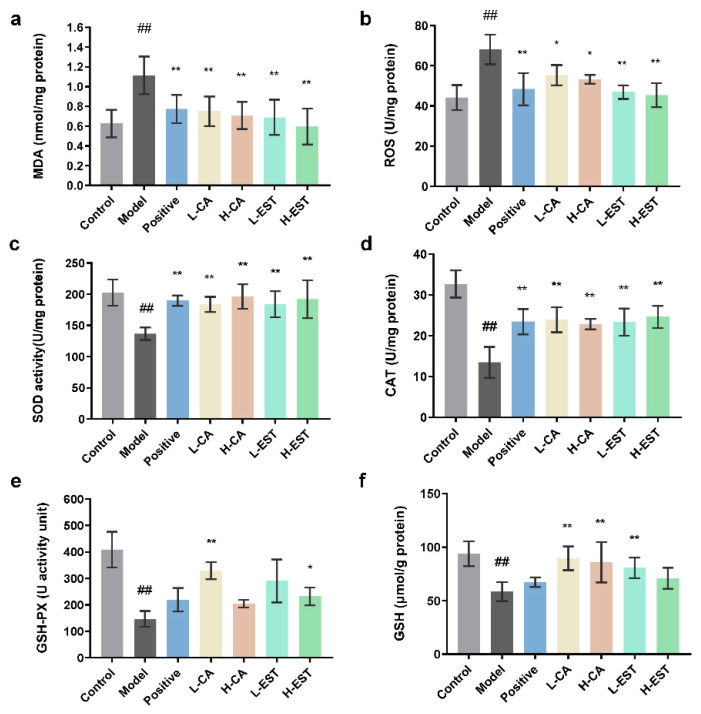
(**a**) MDA, (**b**) ROS level, (**c**) SOD, (**d**) CAT, (**e**) GSH-Px activities, and (**f**) GSH content of liver tissues in each group. Data are expressed as mean ± SD of at least three independent experiments (*n* ≥ 5). ## *p* < 0.01 compared with the control group; * *p* < 0.05, ** *p* < 0.01 compared with the model group.

Oxidative stress is an important factor contributing to CCl_4_-induced liver injury. To evaluate the antioxidant effect of the EST and CA, we analyzed the levels of MDA, ROS, SOD, CAT, GSH-Px, and GSH in the liver tissues. As shown in Figure 3, EST and CA significantly reduced MDA (*p* < 0.01) and ROS (*p* < 0.05) levels and increased those of endogenous antioxidants such as SOD, CAT, GSH-Px, and GSH by varying degrees. These data indicate that both EST and CA restored antioxidant enzyme activity in the liver at different degrees compared with the model group. 

**Figure 4:**
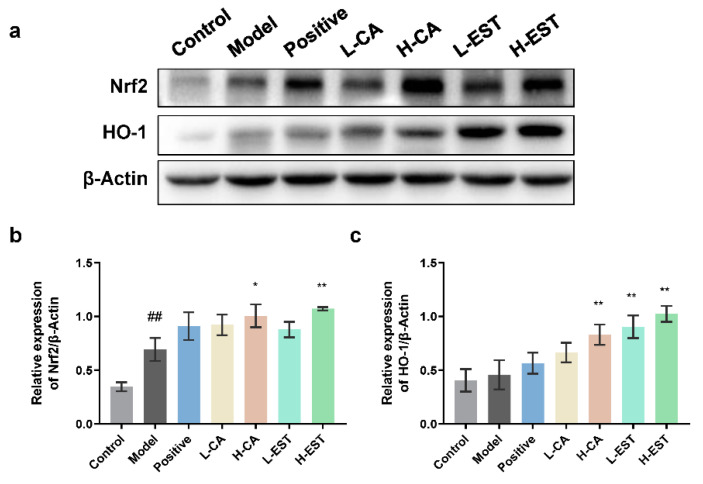
EST and CA decrease the CCl_4_-induced oxidative stress by activating the Nrf2/HO-1. (**a**) Western blotting analysis of Nrf2 and HO-1 expression in liver tissues and densitometric quantification of Nrf 2 (**b**) and HO-1 (**c**) (*n* = 3). Data are expressed as mean ± SD. ## *p* < 0.01 compared with the control group; * *p* < 0.05, ** *p* < 0.01 compared with the model group.

To explore whether the Nrf2/HO-1 signaling pathway was involved in the hepatoprotective effects of EST and CA, we examined the protein expression levels of Nrf2 and HO-1 in liver tissues of different treatment groups by Western blot analysis. As shown in Figure 4, both Nrf2 (*p* < 0.01) and its downstream HO-1 protein expression were increased in the model group compared to the normal group, and the expression was further increased after EST or CA treatment. These results indicate that EST and its main active component CA activate the Nrf2/HO-1 signaling pathway to exert hepatic antioxidant protective effects.

### 3.3. EST and CA Suppress the CC_l4_-Induced Pro-Inflammatory Cytokines Expression via Inhibiting the NF-κB Pathway

**Figure 5:**
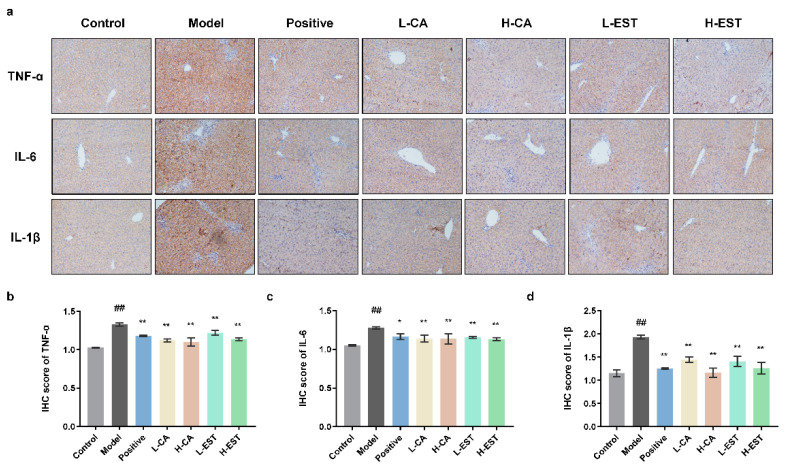
Effects of EST and CA on liver TNF-α, IL-6, and IL-1β expression. (**a**) Immunohistochemical staining of gastric TNF-α, IL-6, and IL-1β. (**b**–**d**) Densitometric quantification (*n* = 3). Data are expressed as mean ± SD. ## *p* < 0.01 compared with the control group; * *p* < 0.05, ** *p* < 0.01 compared with the model group.

In the early stages of oxidative stress, liver immune cells secrete pro-inflammatory factors, such as TNF-α, IL-6, and IL-1β, to trigger inflammatory responses and aggravate liver injury [23]. Immunohistochemical results showed that the protein expression levels of TNF-α, IL-6, and IL-1β were significantly increased in the CCl_4_-induced model group compared with the control group (*p* < 0.01), while both EST and CA could inhibit the expression of these three pro-inflammatory factors in a gradient manner, and the effect was comparable to that of the positive drug, silymarin, indicating that EST and CA could inhibit the expression of CCl_4_-induced pro-inflammatory factors and exerted anti-inflammatory effects (Figure 5).

**Figure 6:**
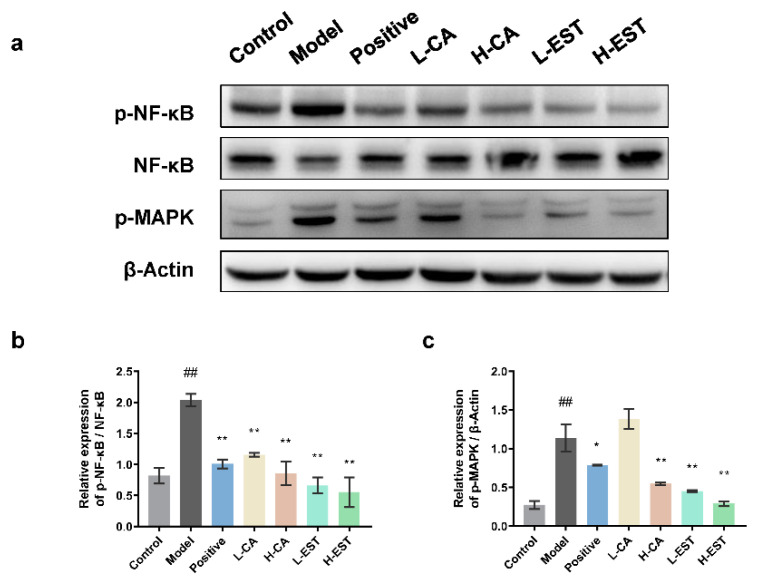
Effects of EST and CA on liver p-NF-κB, NF-κB, p-MAPK expression. (**a**) Western blotting analysis of p-NF-κB, NF-κB, and p-MAPK expression in liver tissues and densitometric quantification of p-NF-κB/NF-κB (**b**) (*n* = 3). (**c**) Western blotting analysis of p-MAPK expression in liver tissues and densitometric quantification of p-MAPK (*n* = 3). Data are expressed as mean ± SD. ## *p* < 0.01 compared with the control group; * *p* < 0.05, ** *p* < 0.01 compared with the model group.

To further examine the effect of EST and CA on inflammatory signaling pathways, the expression of NF-κB and p-MAPK (Figure 6a) in liver tissues, which is vital in inflammatory responses, were determined by Western blot analysis. We found a significant 2-fold upregulation (*p* < 0.01) of p-NF-κB/NF-κB in the liver of the model group compared with the normal group. In contrast, silymarin, EST (200 and 700 mg/kg), and CA (30 and 100 mg/kg) significantly (*p* < 0.01) inhibited the CCl_4_-induced p-NF-κB/NF-κB upregulation (Figure 6b). The effect of different treatment groups of p-MAPK was basically consistent with the ratio of p-NF-κB/NF-κB (Figure 6c). Together, these results suggest that EST and CA alleviate the CCl_4_-induced hepatic inflammatory response by inhibiting the NF-kB signaling pathway and the expression of pro-inflammatory cytokines.

### 3.4. EST and CA Alleviate CCl_4_-Induced Hepatocyte Apoptosis by Regulating the Expression of Bax/Bcl-2

**Figure 7:**
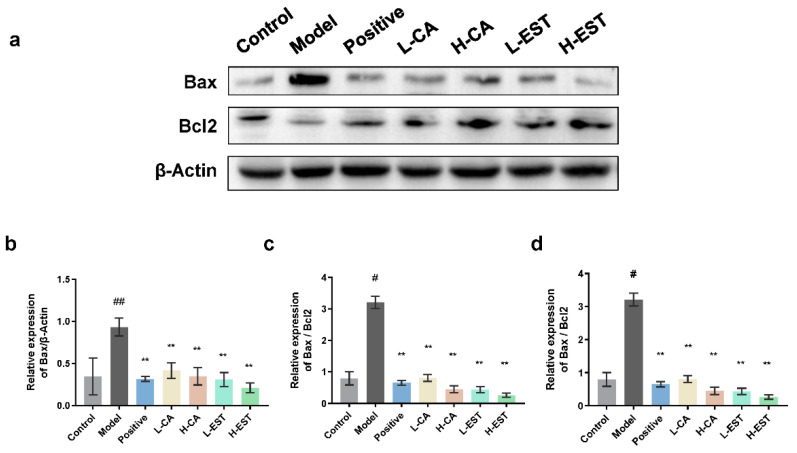
Effects of EST and CA on liver Bax and Bcl2 expression. (**a**) Western blotting analysis of Bax and Bcl2 expression in liver tissues and densitometric quantification of Bax (**b**), Bcl2 (**c**), and Bax/Bcl2 ratio (**d**), (*n* = 3). Data are expressed as mean ± SD. # *p* < 0.05, ## *p* < 0.01 compared with the control group; ** *p* < 0.01 compared with the model group.

Bax, a typical pro-apoptotic factor in cytoplasmic lysis, can be transferred to mitochondria to induce apoptosis, while Bcl-2 can inhibit Bax-induced apoptosis and is an anti-apoptotic factor [24]. Since hepatocyte apoptosis plays an important role in models of liver injury, we investigated the protein expression levels of Bax/Bcl-2 in liver tissues of different groups of mice. As shown in Figure 7, Western blot analysis showed that the expression of the pro-apoptotic protein Bax was significantly upregulated (*p* < 0.01), and the expression of the anti-apoptotic protein Bcl-2 was significantly downregulated (*p* < 0.05) in the model mice compared to the control mice. As with silymarin, CA (30 and 100 mg/kg) and EST (200 and 700 mg/kg) inhibited the CCl_4_-induced increase in Bax expression and decrease in Bcl-2 expression, and the effect was more pronounced in the EST-treated group. We also performed TUNEL analysis to detect the degree of apoptosis in hepatocytes of different treatment groups in Figure S2. These results suggest that EST and CA inhibited the apoptotic signaling pathway by regulating the expression of apoptosis-related proteins Bax and Bcl-2.

## 4. Discussion

Making tea from plants for drinking has been a tradition in China since ancient times. According to the origin of the plants, tea can be divided into traditional tea and non-*Camellia* tea. Traditional tea is a tea beverage made from processed leaves of *Camellia sinensis* plants. In contrast, non-*Camellia* tea refers to tea beverages made from leaves, flowers, and roots of plants that do not belong to *Camellia* [25]. Non-*Camellia* tea usually accumulated certain application experience within a certain region or ethnic group, and, together with traditional tea, forms the colorful tea culture of China [26]. Shibi tea is a kind of non-*Camellia* tea made from the dried leaves of *Adinandra nitida*, which has been reported to have various health benefits such as lowering blood pressure and antibacterial, antioxidant, and analgesic effects, and is commonly used in food and medicine [27,28]. Shibi tea is rich in flavonoids and other active ingredients. The research on the health activity of Shibi tea is mainly focused on the active efficacy of the flavonoid components of Shibi tea, but there is relatively little research on the active efficacy of its major flavonoid monomers, especially CA. In one study, 3T3-L1 cell lines were used as an in vitro model of obesity, combined with nuclear magnetic resonance (NMR) and liquid chromatography (LC)–MS techniques to identify four triterpenoid saponins in Shibi tea that inhibit adipogenesis [29]. In our previous study, we identified CA as the predominant flavonoid in Shibi tea and established a method to prepare CA from Shibi tea [30]. We also investigated for the first time that Shibi tea and CA alleviated alcoholic gastric injury by attenuating HCl/EtOH-induced oxidative stress in the stomach and inhibiting the NF-kB signaling pathway to suppress the expression of inflammatory factors in vivo [17]. However, the mitigating effects of EST and CA on liver injury have not yet been studied.

Liver injury is a common pathological basis of various liver diseases, and clinical study has found that long-term liver injury could lead to mild hepatic inflammation and even liver fibrosis, cirrhosis, and hepatocellular carcinoma [31,32]. Liver injury is still a global health issue, and the hunt for novel hepatoprotective methods is extremely important. Experimental animal models are useful models for the study of drug-induced liver injury and its pathogenesis. Among them, acetaminophen (Acetaminophen, APAP) and CCl_4_ are the two most common model inducers in the study of endogenous drug-induced liver injury [33]. Oxidative stress pathways, inflammatory cytokines, and apoptosis are the main regulatory targets of liver injury [34]. When liver injury occurs, hepatocytes and endothelial cells are damaged, hepatic Kupffer cells (KC) and hepatic stellate cells (HSC) are activated, and inflammatory factors accumulate in the liver. As the therapeutic effects of flavonoids on liver injury have been studied recently, the significant protective effects of flavonoids on liver injury have been confirmed. Flavonoids, such as silymarin [35,36], can reduce the production of oxidative free radicals and increase the activity of antioxidant enzymes, effectively protecting the liver from oxidative damage. Studies have shown that flavonoids protect hepatocytes and endothelial cells from oxidative stress and inhibit the proliferation and activation of activated HSC and promote their apoptosis; in addition, flavonoids inhibit TNF-α production and KC activation and reduce the accumulation of inflammatory factors [37]. In this study, we demonstrated that the EST (200 and 700 mg/kg) and its major flavonoid CA (30 and 100 mg/kg) significantly ameliorated CCl_4_-induced acute liver injury (Figure 1 and Figure 2) in a mouse model with intraperitoneal injection of CCl_4_, using silymarin as a positive drug control, and that its hepatoprotective mechanism was related to the antioxidant, anti-inflammatory, and anti-apoptotic effects of EST and CA. Given that CA is the main flavonoid component of EST and the hepatoprotective effect is comparable to that of EST, we propose that CA is potentially the main active substance contributing to the hepatoprotective effect in EST. 

Oxidative stress is an important cause of liver injury, and the metabolites of CCl_4_, CCl3- and CCl3OO-, both of which can cause oxidative stress in hepatocytes and consequently hepatocellular injury [38]. Flavonoids improve oxidative stress by activating the Nrf2/HO-1 pathway, and Nrf2 is a key transcription factor that upregulates the antioxidant gene HO-1 [39]. Plant-derived flavonoids such as cyanidin flavonoids [40] and alpinetin [41] have also been reported to effectively alleviate CCl_4_-induced acute liver injury in mice by modulating the Nrf2 signaling pathway, reducing ROS and MDA levels, and increasing antioxidant enzyme activity. In this study, we found that EST and CA increased the activities of antioxidant enzymes SOD, CAT, and GSH-Px in liver tissues; increased the level of antioxidant GSH; inhibited the CCl_4_-induced increase in MDA and ROS oxidative stress levels (Figure 3); and significantly activated antioxidant pathway targets Nrf2 and HO-1 to perform antioxidant effects (Figure 4).

Abnormal expression of inflammatory cytokines, which mediate the interference of various immune cells, can directly affect the immune response, and occur frequently during liver injury. Morin [42] significantly inhibited lipopolysaccharide (LPS)-induced production of serum AST, ALT, IL-6, and TNF-α, which could exert antioxidant and anti-inflammatory protective effects by activating the Nrf2 antioxidant signaling pathway and inhibiting the NF-κB inflammatory pathway. Breviscapine [8] inhibited significantly elevated levels of serum TNF-α, IL-6, IL-1β, and monocyte chemotactic protein-1 (MCP-1) in a CCl_4_ model group of mice and suppressed the expression of downstream IκBα and NF-κB inflammatory pathways, acting as hepatoprotective agents. Similarly, EST and CA significantly inhibited CCl_4_-induced liver injury by targeting the MAPK and NF-κB pathways (Figure 6) and the downstream inflammatory cytokines, such as TNF-α, IL-6, and IL-1β (Figure 5).

Flavonoids can also reduce liver injury by regulating apoptosis; for example, dihydromyricetin significantly inhibited the expression of the pro-apoptotic protein Bax and upregulated the expression of the anti-apoptotic protein Bcl-2, thereby inhibiting chronic liver injury [43]. Baicalin played an important hepatic repair role in oxidative stress-induced liver injury by regulating mitochondria-related apoptosis [44]. Wogonin significantly increased the Bax/Bcl-2 ratio in T6 cells and regulated the activation and apoptosis of hepatic stellate cells to reduce liver fibrosis [45]. We found that EST and CA regulate apoptosis by regulating Bax/Bcl-2 expression to alleviate CCl_4_-induced liver injury (Figure 7).

In this study, we found that for the CA and EST treatment groups, the high-dose group generally showed better results than the low-dose group, but did not reach a significant difference in some of the results. Therefore, the current study provides a basis for further research on the active dosage and underlying mechanisms of CA and EST hepatoprotective effects. In addition, we used 30 and 100 mg/kg CA treatments and 200 and 700 mg/kg EST treatments, which are much lower than the reported flavonoid monomer concentrations (>500 mg/kg) that cause toxicity or side effects. Orally administrated dosages of 200–700 mg/kg EST are in accordance with the dosage taken by drinking tea (3–10 g) per day. There were no effects of CA and EST on animal behavior during the experiment and no significant visual damage to other organs of mice in all groups.

## 5. Conclusions

Our study demonstrates that EST and its major flavonoid component, CA, can effectively alleviate CCl_4_-induced acute liver injury in mice. More importantly, the hepatoprotective effects of EST and CA are primarily attributed to the reduction of oxidative stress, inhibition of inflammation, and regulation of apoptosis. Additionally, these results provide a theoretical basis for the further investigation of EST and CA as potential agents for the treatment and prevention of liver disease.

## Data Availability

Not applicable.

## Supplementary Materials

The following supporting information can be downloaded at: https://www.mdpi.com/article/10.3390/nu14153037/s1, Figure S1: Masson trichrome staining of the liver sections of different groups; Figure S2. Histological changes (TUNEL assay) in the liver of different groups.
