# Supplementary material for: Shibi Tea (Adinandra nitida) and Camellianin A Alleviate CCl4-Induced Liver Injury in C57BL-6J Mice by Attenuation of Oxidative Stress, Inflammation, and Apoptosis
Source: Nutrients. 2022 Jul 24;14(15):3037. doi: 10.3390/nu14153037 (PMC9332116; doi:10.3390/nu14153037)
Supplement: Supplementary file 1 [file nutrients-14-03037-s001.zip › nutrients-1768786-supplementary.pdf]

nutrients

MDPI

Supplementary Material

Shibi Tea (*Adinandra nitida*) and Camellianin A Alleviate CCl_4-Induced Liver Injury in C57BL-6J Mice by Attenuation of Oxidative Stress, Inflammation, and Apoptosis

Ruohong Chen [1,#], Yingyi Lian [2,#], Shuai Wen [1], Qiuhua Li [1], Lingli Sun [1], Xingfei Lai [1], Zhenbiao Zhang [1], Junquan Zhu [3], Linsong Tang [4], Ji Xuan [5], Erdong Yuan [2,*], and Shili Sun [1,*]

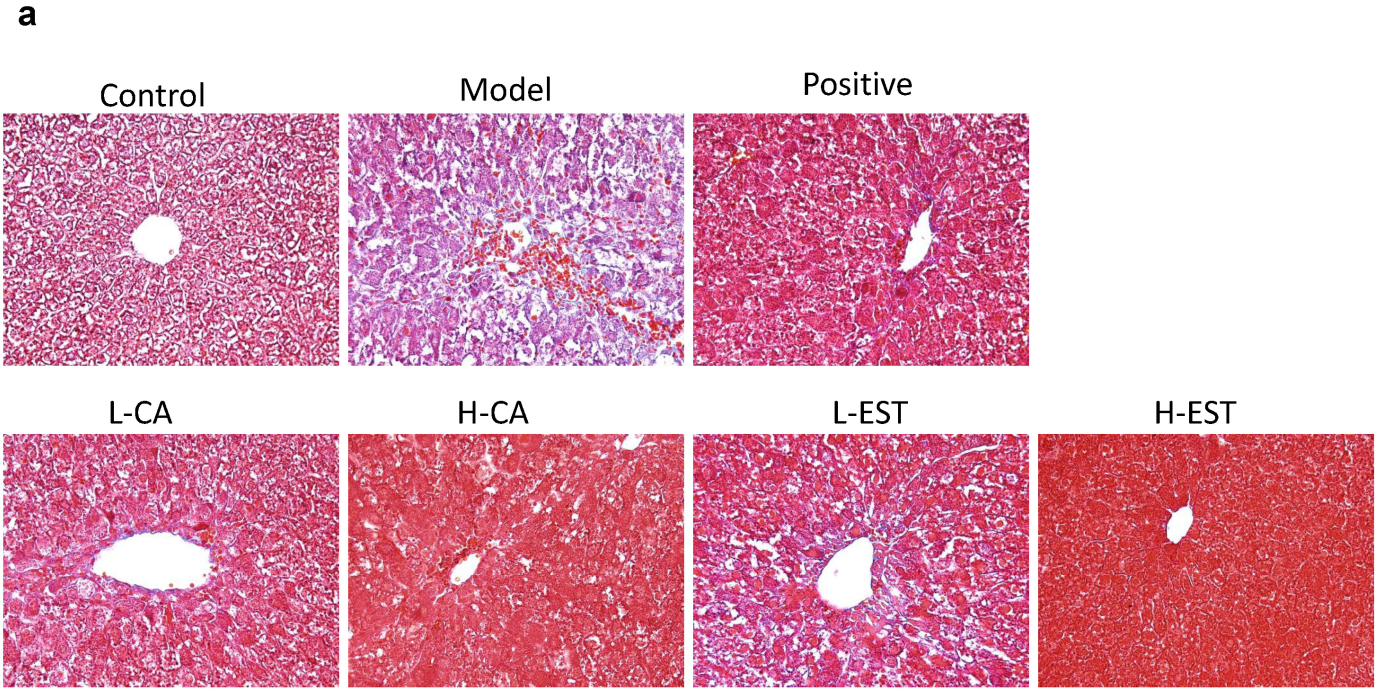

Figure S1. Masson' trichrome staining of the liver sections of different groups.

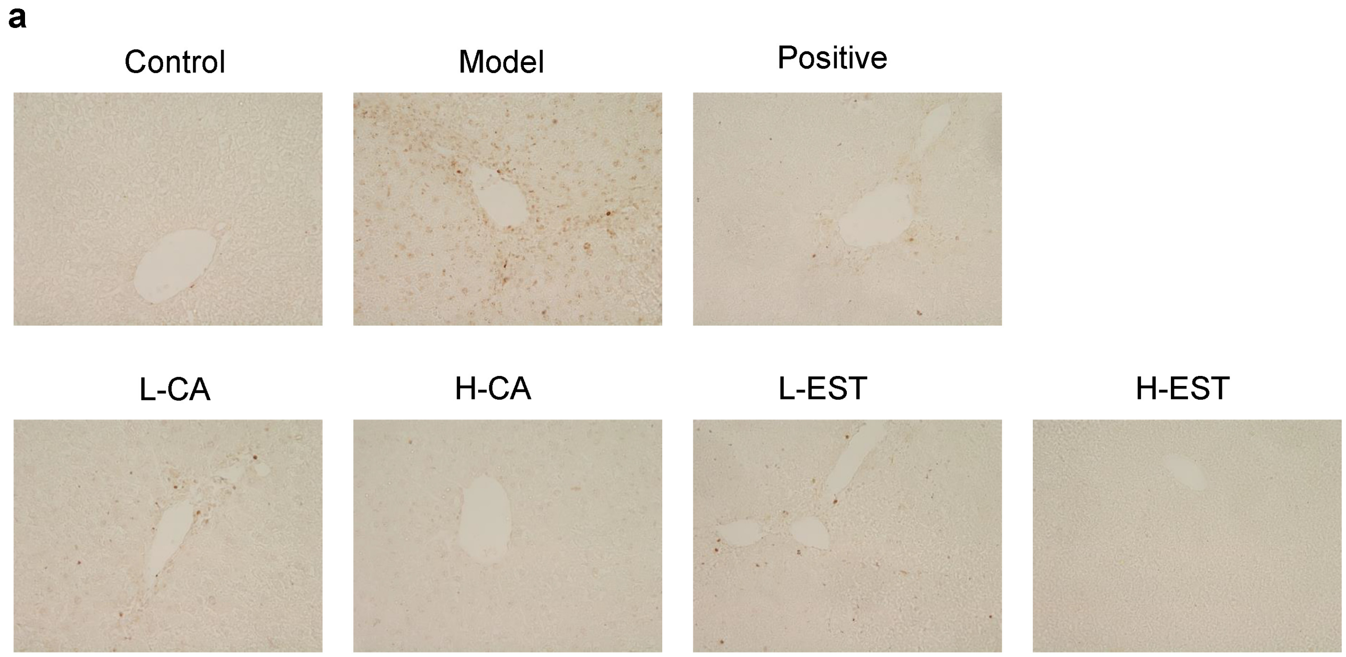

Figure S2. Histological changes (TUNEL assay) in the liver of different groups.
